# Supplementary material for: Content-rich biological network constructed by mining PubMed abstracts
Source: BMC Bioinformatics. 2004 Oct 8;5:147. doi: 10.1186/1471-2105-5-147 (PMC528731; doi:10.1186/1471-2105-5-147)
Supplement: Additional File 5 — The original Chilibot query results of the term "long-term potentiation (LTP)" and 22 other terms, limiting the latest references analyzed to the years 1990, 1995, 2000, and 2004. [file 1471-2105-5-147-S5.bz2 › chilibotAdditionalFile5/ltp2004/html/TRKB_ERK.html]

 


 **TRKB** and **ERK** 
  
Found 22 abstracts in PubMed,  **22 abstracts were retrieved and analyzed**.  


---

 Search Google  |
 PDF files only 
|  EDU domain only 

---

**Interactive relationship** (e.g. stimulation, inhibition, etc)

- Finally, we found that sequestering BDNF in vivo with a  **TrkB**  IgG fusion molecule significantly reduced the activation of  **ERK**  evoked by noxious stimulation.  Ref: 12504600 Mol Cell Neurosci, 2002

- :-)

  **Parallel relationship** (e.g. studied together, co-existance, homology, etc.)

  - These data suggest that BDNF, once released with activity from primary afferent nociceptors, exerts a neuromodulatory role in pain processing through stimulation of postsynaptic  **TrkB**  receptors and subsequent activation of  **ERK** .  Ref: 12504600 Mol Cell Neurosci, 2002
  - In summary, we demonstrate that  **TrkB**  mediated activation of the  **ERK**  pathway is preferentially required for BDNF induction of NPY produced and secreted but not for the induction of the expression of neurite bearing NPY neurons.  Ref: 11689163 Brain Res, 2001
  - Glutamate release was also enhanced, and the data suggest that this may be a consequence of increased activation of  **TrkB**  and  **ERK** .  Ref: 12049326 Behav Neurosci, 2002
  - In both cases, KCl induced brain derived neurotrophic factor release was increased, and this was accompanied by increased phosphorylation of  **TrkB**  and the mitogen activated protein kinase,  **ERK** .  Ref: 12049326 Behav Neurosci, 2002
  - Our results demonstrate that the signal transduction pathways through TRK A and TRK B  [ **TRKB** ]  in MP N TS cells are functional and similar, and the main downstream signaling pathways from the three neurotrophins are mitogen activated protein kinase cascades through Shc, activated Ras,  **ERK**  1 and  **ERK**  2, and the transduction pathway through PLC gamma1.  Ref: 11223544 Jpn J Cancer Res, 2001
  - Long term potentiation and spatial learning are associated with increased phosphorylation of  **TrkB**  and extracellular signal regulated kinase  **ERK**  in the dentate gyrus evidence for a role for brain derived neurotrophic factor.  Ref: 12049326 Behav Neurosci, 2002
  - BDNF signaling via  **TrkB**  receptors but not SP signaling via NK 1 were also involved in  **ERK**  recruitment.  Ref: 14572451 Mol Cell Neurosci, 2003
